# Cow Placenta Peptides Ameliorate D-Galactose-Induced Intestinal Barrier Damage by Regulating TLR/NF-κB Pathway

**DOI:** 10.3390/vetsci12030229

**Published:** 2025-03-03

**Authors:** Yuquan Zhao, Zhi Zeng, Weijian Zheng, Zeru Zhang, Hanwen Zhang, Yuxin Luo, Kunshan Zhao, Yuyan Ding, Wei Lu, Fuxing Hao, Yixin Huang, Liuhong Shen

**Affiliations:** 1 The Key Laboratory of Animal Disease and Human Health of Sichuan Province, The Medical Research Center for Cow Disease, College of Veterinary Medicine, Sichuan Agricultural University, Chengdu 611130, China; zhaoyuquan@stu.sicau.edu.cn (Y.Z.); 2023303092@stu.sicau.edu.cn (Z.Z.); zhengweijian@stu.sicau.edu.cn (W.Z.); zhangzr@stu.sicau.edu.cn (Z.Z.); 2023203075@stu.sicau.edu.cn (H.Z.); llyuxx@stu.sicau.edu.cn (Y.L.); zhaokunshan@stu.sicau.edu.cn (K.Z.); dingyuyan@stu.sicau.edu.cn (Y.D.); 2 Jiangsu Agri-Animal Husbandry Vocational College, Taizhou 225300, China; 2002010183@jsahvc.edu.cn (W.L.); vethfx@163.com (F.H.)

**Keywords:** aging, intestinal barrier, cow placenta peptides, D-galactose, TLR4/NF-κB

## Abstract

This study investigated the protective effects and mechanisms of cow placenta peptides (CPP) on intestinal barrier damage in aging model mice. Forty-eight male ICR mice were assigned to four groups: a control group (N), an aging model group (M), a CPP treatment group (T), and a vitamin C treatment group (P). Groups T and P received oral administration of CPP (2000 mg/kg/day) and vitamin C (100 mg/kg/day), respectively, while groups M, T, and P were subjected to intraperitoneal injections of D-galactose (D-gal) (300 mg/kg/day). Group N received an equivalent volume of normal saline via intraperitoneal injection. Treatments were administered once daily for 8 weeks. The results demonstrated that CPP significantly alleviated D-galactose-induced intestinal structural damage, increasing the villus height-to-crypt depth ratio and reducing serum diamine oxidase (DAO) and lipopolysaccharide (LPS) levels. CPP notably alleviated intestinal oxidative stress and inflammation, restored tight junction expression, and enhanced intestinal barrier integrity. Transcriptome sequencing identified 1396 DEGs associated with CPP’s effects, highlighting *TLR4*, *IL-1β*, and *Mmp9* as core regulatory genes through protein–protein interaction network analysis. Kyoto Encyclopedia of Genes and Genomes and Gene Ontology enrichment analyses implicated the TLR4/NF-κB signaling pathway, which was further validated. Western blotting confirmed that CPP significantly down-regulated TLR4, IKKβ, and p-NF-κB p65 protein expression in the intestines of aging mice. In conclusion, CPP effectively alleviates D-gal-induced intestinal barrier damage in aging mice by enhancing antioxidant defense and inhibiting the TLR4/NF-κB signaling pathway, thereby diminishing inflammation and protecting intestinal barrier integrity.

## Simple Summary

The intestinal barrier is crucial for maintaining overall health, but it can be compromised by aging and oxidative stress. D-galactose, commonly used to induce aging in research models, causes intestinal barrier damage, oxidative stress, and inflammation. Cow placenta peptides (CPP) have demonstrated antioxidant and anti-inflammatory properties, making them a promising therapeutic option. In this study, CPP effectively alleviated D-galactose-induced intestinal barrier damage by enhancing antioxidant defenses, reducing inflammation, and restoring tight junctions essential for barrier integrity. Moreover, CPP regulated key genes and pathways, notably the TLR4/NF-κB signaling pathway, which is crucial for gut health. These findings suggest that CPP could serve as a potential treatment approach for safeguarding intestinal barrier integrity during aging.

## 1. Introduction

Aging is an inevitable physiological process characterized by progressive tissue and organ damage and functional decline [1]. The free radical theory posits that the efficiency of free-radical elimination decreases with age, leading to an imbalance between oxidation and antioxidation [2]. Excessive reactive oxygen species (ROS) contribute to cellular structural and functional impairment, accelerating the dysfunction of tissues and organs during the aging process [3,4,5,6]. The intestine, as the primary digestive and largest immune organ, performs a vital function in nutrient assimilation and acts as a barrier against pathogenic microbial invasion [7]. Due to its location at the interface between the body and the intestinal lumen, the intestinal epithelium is particularly vulnerable to harmful substances such as dietary toxins, drugs, and environmental pollutants, which stimulate excessive ROS production, triggering oxidative stress and inflammatory responses [8]. These stressors accelerate intestinal aging, leading to epithelial cell damage, reduced tight junction (TJ) expression, and compromised barrier integrity, ultimately impairing digestive and absorptive functions [9]. A weakened intestinal barrier permits the translocation of pathogenic microorganisms and antigens, inducing low-grade systemic inflammation [10]. Consequently, mitigating the detrimental effects of aging on intestinal barrier integrity is crucial for enhancing animal production performance and welfare.

Currently, most anti-aging pharmacological agents, such as chemically synthesized metformin, rapamycin, aspirin, simvastatin, and vitamin C (Vit C), achieve targeted therapeutic effects but are often associated with significant side effects and dependency risks, making them unsuitable for long-term use [11]. Thus, there is an urgent need to explore natural, non-toxic, and resistance-free anti-aging alternatives. Peptide-based compounds are particularly promising due to their excellent safety profile, stability, bioavailability, and nutritional benefits [12]. These peptides can alleviate oxidative damage, reduce inflammation, and enhance intestinal barrier function, thereby contributing to intestinal homeostasis. For instance, oyster peptides enhance antioxidant enzyme activity in the intestine, mitigating oxidative stress-induced intestinal damage [13], while sea cucumber peptides upregulate TJs, enhancing intestinal barrier integrity [14]. Cow placenta, a rich natural resource, remains underutilized despite its potential. Previous research by our team optimized extraction conditions for cow placenta peptides (CPP) and characterized them as small bioactive peptides via mass spectrometry. These peptides exhibit potent antioxidant, anti-inflammatory, and cell proliferation-promoting properties, mitigating aging-related damage to the skin [15] and liver [16]. Moreover, CPP has demonstrated protective effects against intestinal tissue damage caused by immunosuppression, enhancing barrier function [17]. Therefore, it is hypothesized that CPP may significantly protect against aging-related intestinal barrier damage. D-galactose (D-gal) serves as an effective inducer of aging by triggering oxidative stress, inflammatory responses, and advanced glycation end products (AGEs) [18]. A model of D-gal-induced senescence closely mimics natural aging in terms of morphological and functional changes in tissues and cytokine changes, making it a widely used model in aging research [19]. This study aims to investigate the preventive effects and mechanisms of CPP on intestinal barrier damage in D-gal-induced senescent mice, providing scientific evidence for developing anti-aging interventions targeting intestinal barrier dysfunction.

## 2. Materials and Methods

### 2.1. Preparation of CPP

CPP was prepared following the protocols established in our previous study [20]. The cow placenta underwent enzymatic digestion with papain, following which, the supernatant was separated by centrifugation, resulting in the acquisition of CPP through freeze-drying. CPP consists of 128 sourced peptides, all possessing relative molecular weights under 3000 Da and peptide lengths primarily between 7 and 25 amino acids [16].

### 2.2. Animals and Treatment

Forty-two male ICR mice, aged 8 weeks old (SCXK (jing) 2019-0010, SPF (Beijing) biotechnology Co., Ltd., Beijing, China), were randomly assigned to four groups following a 1-week acclimatization period: the blank control group (N), the aging model group (M), the CPP treatment group (T), and the vitamin C positive control group (P) (*n* = 12). Groups M, T, and P were administered intraperitoneal injections of D-gal (300 mg/kg/day), while the group N received an equivalent volume of normal saline. Concurrently, groups T and P were administered CPP (2000 mg/kg/day) [15,16] and Vit C (100 mg/kg/day) via gavage, respectively, while the groups N and M received equal volumes of normal saline. All treatments were administered once daily for a duration of 8 weeks. Body weights were measured weekly to adjust dosing.

Modeling criteria included weight reduction in mice, shrinkage of the intestinal villi, disorganized arrangement, epithelial loss [21], and elevated levels of intestinal malondialdehyde (MDA), tumor necrosis factor-alpha (TNF-α), AGEs, interleukin (IL)-1β, and IL-6 [22]. Additionally, impaired intestinal barrier was indicated by heightened permeability and reduced expression of zonula occludens-1 (ZO-1), Occludin, and Claudin-1 [23].

### 2.3. Sample Collection

After the final administration, the mice were fasted for 12 h and then anesthetized by isoflurane inhalation for blood sampling from the eyeballs. Blood was permitted to rest for 30 min and thereafter centrifuged at 920× *g* for 5 min at 4 °C to isolate serum. The duodenum, jejunum, and ileum were removed through laparotomy. The intestinal segments were rinsed with PBS and subsequently immersed in 4% paraformaldehyde fixing solution, RNAstore reagent (DP408-02, Tiangen Biotech Co., Ltd., Beijing, China), and cryopreservation tubes for future tests, respectively.

### 2.4. H&E and PAS Staining of Intestines

Intestinal tissues were paraffin embedded after fixation in 4% paraformaldehyde and sectioned into 4 μm slices. Sections were deparaffinized using xylene, hydrated with alcohol, and subsequently stained with hematoxylin and eosin (H&E) and periodic acid–Schiff (PAS). Microscopic photographs were captured using an Olympus microscope (Tokyo, Japan), and the villus height (V) and crypt depth (C) were quantified with ImageJ-v1.54 software.

### 2.5. Detection of Serum DAO and LPS Levels

Serum levels of diamine oxidase (DAO) and lipopolysaccharide (LPS) were quantified utilizing kits from Shanghai Enzyme Link Biotechnology Co., Ltd. (Shanghai, China).

### 2.6. Intestinal Oxidation, Aging Markers, and TJ Assays

Intestinal tissue was weighed and mixed with PBS at a 1:9 weight-to-volume ratio and then thoroughly homogenized at 4 °C using a tissue grinder (Meibi Instruments Co., Ltd., Jiaxing, China). The homogenate underwent centrifugation at 4600× *g* for 20 min at 4 °C, after which the supernatant was extracted from the centrifuge tube. The levels of MDA, ROS, 8-hydroxy-2′-deoxyguanosine (8-OHdG), senescence-associated β-galactosidase (SA-β-Gal), AGEs, ZO-1, Claudin-1, and Occludin in the supernatant were measured utilizing commercially available enzyme-linked immunosorbent assay (ELISA) kits (Shanghai Enzyme Link Biotechnology Co., Ltd., Shanghai, China) in accordance with the manufacturer’s guidelines. Briefly, for each assay, standards and samples were introduced into microtiter wells pre-coated with primary antibody, followed by the addition of horseradish peroxidase (HRP)-conjugated secondary antibody, which was incubated for 60 min at 37 °C. After washing using the supplied buffer, the reaction was conducted utilizing tetramethylbenzidine (TMB) substrate, and the absorbance was determined at 450 nm. ELISACalc-v0.1 software was utilized to derive the quadratic regression equation and determine the concentration of the test sample.

### 2.7. Detection of Genes Related to Intestinal Oxidation, Inflammation, and TJs

Total RNA was extracted from each intestinal tissue segment with the RNA Easy Fast Tissue Kit (DP451, Tiangen Biotech Co., Ltd., Beijing, China), and the RNA concentration and purity were assessed with a Nanodrop 2000 spectrophotometer (Thermo Fisher Scientific, Waltham, MA, USA). The isolated mRNA was reverse-transcribed into cDNA utilizing FastKing gDNA Dispelling RT SuperMix (KR118, Tiangen Biotech Co., Ltd., Beijing, China). Amplification reactions were conducted with a Bio-Rad apparatus (Hercules, CA, USA) utilizing FastReal qPCR PreMix (FP217, Tiangen Biotech Co., Ltd., Beijing, China). The data were analyzed utilizing the 2^−ΔΔCT^ method, with relative expression data for group T expressed as log2 [24]. Primer sequences are provided in the Supplementary Materials.

### 2.8. Immunofluorescence and Immunohistochemical Staining of Intestines

Immunofluorescence: After deparaffinizing the paraffin sections in water, they were subjected to boiling in citrate buffer for 10 min. Thereafter, primary antibodies were administered, and the sections were incubated at 4 °C for 12 h. Following the washing procedure, secondary antibodies were administered and incubated at ambient temperature for 50 min in the absence of light. Nuclei were stained with DAPI. Immunohistochemistry: Paraffin sections underwent treatment with a 3% H_2_O_2_ solution for 30 min following antigen retrieval. Sections were subsequently blocked with 3% BSA for 30 min and treated with primary antibodies overnight at 4 °C. Following the washing procedure, secondary antibodies were administered and incubated for 60 min at ambient temperature. Antibody information is supplied in the Supplementary Materials.

### 2.9. RNA Sequencing of Intestines

Total RNA was extracted from mouse intestinal tissue utilizing TRIzol reagent (15596026CN, Thermo Fisher Scientific, Waltham, MA, USA). The concentration and purity of the collected RNA were quantified utilizing a Nanodrop 2000 (Thermo Fisher Scientific, Waltham, MA, USA) instrument, and RNA integrity was assessed with a DYY-6C analyzer (Beijing Liuyi Instrument Factory, Beijing, China). Additionally, the RNA quality number was determined with an Agilent 5300 analyzer (Agilent, Palo Alto, CA, USA). cDNA libraries were then constructed using an RNA Sample Preparation Kit (20040532, Illumina, San Diego, CA, USA). RNA sequencing was conducted utilizing the Illumina NovaSeq X Plus platform (Illumina, San Diego, CA, USA), and raw data were processed and analyzed on the Majorbio Cloud Platform (https://cloud.majorbio.com). Differentially expressed genes (DEGs) between groups were identified with criteria of |log2FC| ≥ 1 and *p* < 0.05. These DEGs were analyzed using STRING 11.0 to identify targets with a confidence score > 0.4, and the data were visualized using Cytoscape 3.9.1. A protein–protein interaction (PPI) network was constructed based on node degree values.

### 2.10. Detection of Expression of Proteins Related to Key Signaling Pathways in Intestines

Total protein was isolated from mice intestinal tissue utilizing RIPA lysis buffer. Protein concentration was measured utilizing a BCA Protein Assay Kit (PC0020, Solarbio Life Sciences, Beijing, China), and samples were standardized to uniform concentrations. Proteins were fractionated using 12.5% SDS-PAGE and then transferred to PVDF membranes. The membranes were obstructed using Tris-buffered saline with 1% Tween-20 and 5% skim milk. Subsequently, they were treated overnight at 4 °C with the relevant primary antibodies, followed by a 2 h incubation with secondary antibodies at room temperature. Images were obtained utilizing the iBright 1500 imaging system (Thermo Fisher Scientific, Waltham, MA, USA), and density analysis was conducted with ImageJ software. Information on antibodies is provided in the Supplemental Materials.

### 2.11. Statistical Analysis

Statistical analysis was conducted using SPSS 26.0 software (IBM, Chicago, IL, USA). A one-way analysis of variance was used to assess differences among experimental groups, followed by a Tukey’s post hoc analysis. Data are presented as mean ± SEM, with statistical significance established at *p* < 0.05.

## 3. Results

### 3.1. Body Weight Changes

**Figure 1:**
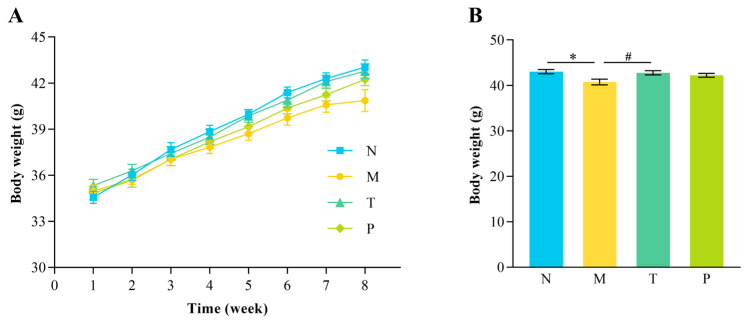
Variation in body weight of mice. (**A**) Growth curves of mice; (**B**) final body weights of mice. “^#^” denotes *p* < 0.05; “*” denotes *p* < 0.05 (*n* = 12).

The body weight gain rate in group M began to significantly decrease compared to that in group N starting from week 4 (*p* < 0.05), whereas group T exhibited a trend comparable to that in group N (Figure 1A). As shown in Figure 1B, the final body weight of group M was notably lower compared to that of group N (*p* < 0.05). In contrast, the final body weight in group T was notably higher than that in group M (*p* < 0.05).

### 3.2. Histological and PAS Staining of Intestines

**Figure 2:**
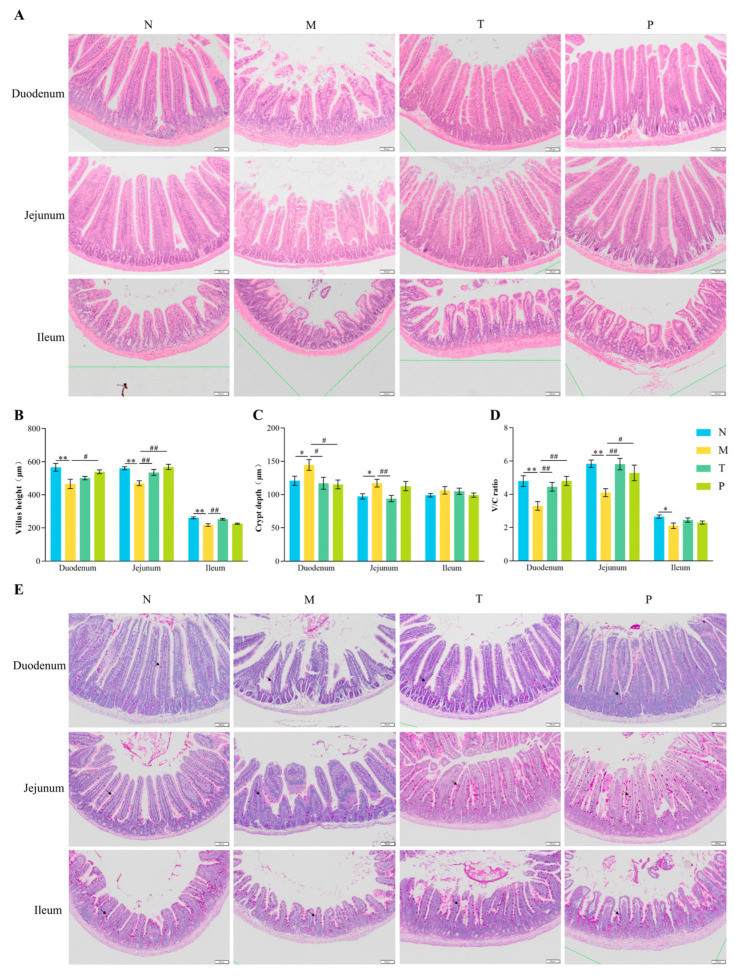
Detection results of tissue structure in the intestine of mice. (**A**) Representative image of an H&E-stained intestinal tissue sample (scale: 100 µm); (**B**) villus height; (**C**) crypt depth; (**D**) ratio of villus height to crypt depth; (**E**) representative images of PAS-stained intestinal tissue samples (scale: 100 µm). “→”: goblet cell; “^#^” denotes *p* < 0.05; “^##^” denotes *p* < 0.01; “*” denotes *p* < 0.05; “**” denotes *p* < 0.01 (*n* = 10).

H&E staining results (Figure 2A) showed that the intestinal mucosal epithelium in group N displayed an intact structure, abundant intestinal glands, and well-aligned villi. In contrast, group M exhibited villus atrophy, disorganized villi, epithelial loss, and varying degrees of vacuolization. The intestinal structure in groups T and P was largely restored, with villi appearing intact, orderly, and smooth, resembling those of group N. Additionally, in contrast to group N, group M exhibited markedly diminished villus height/crypt depth (V/C) ratios across all intestinal segments (*p* < 0.05 or *p* < 0.01), with crypt depth in the duodenum and jejunum significantly elevated (*p* < 0.05). In comparison to group M, group T exhibited markedly longer villi in the jejunum and ileum, an elevated V/C ratio in the duodenum and jejunum (*p* < 0.01), and a significantly decreased crypt depth in these regions (*p* < 0.05 or *p* < 0.01). Similarly, group P showed notable improvements in villus height and V/C ratios in the duodenum and jejunum compared to group M (*p* < 0.05 or *p* < 0.01), alongside a considerably diminished crypt depth in the duodenum (*p* < 0.05). PAS staining (Figure 2E) indicated a reduction in goblet cell numbers in the duodenum and jejunum of group M compared to those in group N, while groups T and P displayed varying degrees of recovery with increased goblet cell numbers.

### 3.3. Serum DAO and LPS Assay

**Figure 3:**
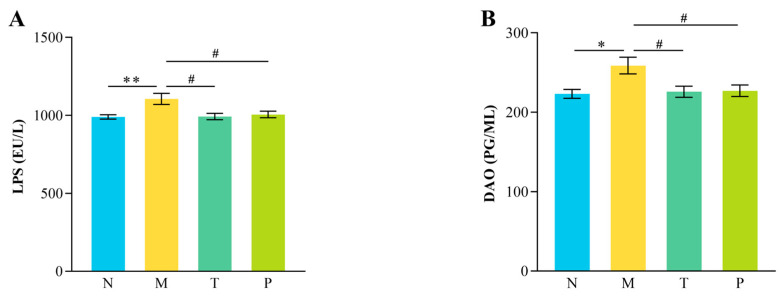
Serum-related indicators of intestinal permeability. (**A**) Lipopolysaccharide levels; (**B**) diamine oxidase activity. “^#^” denotes *p* < 0.05; “*” denotes *p* < 0.05; “**” denotes *p* < 0.01 (*n* = 12).

As illustrated in Figure 3, group M exhibited significantly elevated serum LPS levels compared to group N (*p* < 0.01), as well as higher DAO activity (*p* < 0.05). Conversely, serum DAO activity and LPS levels in groups T and P were notably lower than those in group M (*p* < 0.05), with no significant differences compared to group N (*p* > 0.05).

### 3.4. TJ Gene Expression in Intestinal Segments

**Figure 4:**
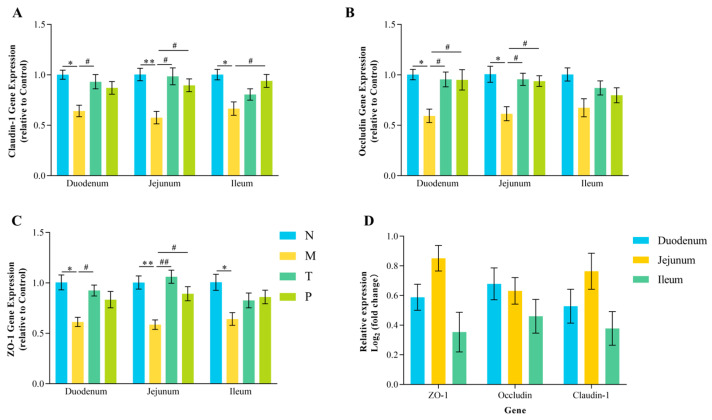
Expression of TJ genes in the mouse intestine. (**A**) Claudin-1; (**B**) Occludin; (**C**) ZO-1; (**D**) relative expression of TJ-related genes in three segments of the small intestine. “^#^” denotes *p* < 0.05; “^##^” denotes *p* < 0.01; “*” denotes *p* < 0.05; “**” denotes *p* < 0.01 (*n* = 3).

Figure 4 demonstrates that the expression levels of *ZO-1* and *Claudin-1* were notably diminished in all three intestinal segments of group M in comparison to those in group N (*p* < 0.05 or *p* < 0.01). Similarly, *Occludin* expression was markedly reduced in the duodenum and jejunum of group M (*p* < 0.05). In contrast, the expression levels of *ZO-1*, *Claudin-1*, and *Occludin* were markedly elevated in the duodenum and jejunum of group T relative to those in group M (*p* < 0.05 or *p* < 0.01). In comparison to that in group M, the expression of *Occludin* in the duodenum of group P was markedly elevated (*p* < 0.05). Additionally, in the jejunum, the expression levels of *ZO-1*, *Occludin*, and *Claudin-1* were notably higher in group P (*p* < 0.05). In the ileum, group P exhibited a markedly elevated level of *Claudin-1* expression compared to that in group M (*p* < 0.05). Within group T, the expression of *ZO-1* and *Claudin-1* was highest in the jejunum, whereas *Occludin* expression was most prominent in the duodenum (Figure 4D). These results demonstrate that CPP exhibits the most pronounced effect on improving the expression of TJs in the jejunum, which will be the focus of the subsequent investigations.

### 3.5. Validation of TJs in the Jejunum

**Figure 5:**
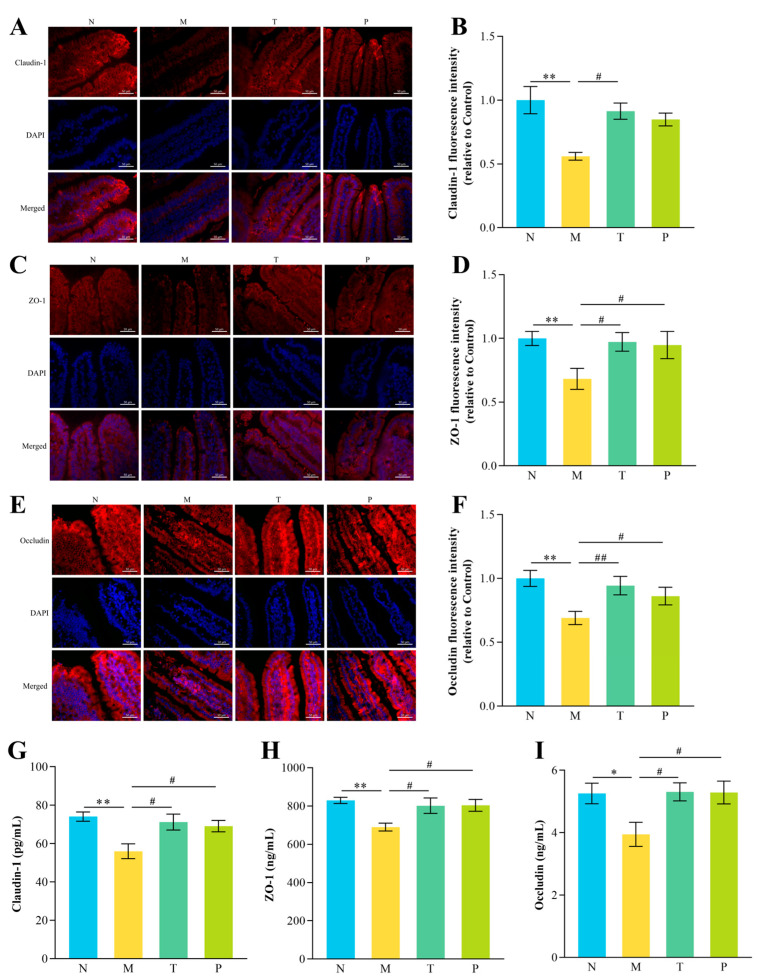
Results of mouse jejunal TJ-related protein detection. (**A**) Jejunal Claudin-1 fluorescence images (original magnification, 400×); (**B**) jejunal Claudin-1 fluorescence intensity; (**C**) jejunal ZO-1 fluorescence images; (**D**) jejunal ZO-1 fluorescence; (**E**) jejunal Occludin fluorescence images; (**F**) jejunal Occludin fluorescence; (**G**) jejunal Claudin-1 protein expression levels; (**H**) jejunal ZO-1 protein expression levels; (**I**) jejunal Occludin protein expression levels. “^#^” denotes *p* < 0.05; “^##^” denotes *p* < 0.01; “*” denotes *p* < 0.05; “**” denotes *p* < 0.01 (*n* = 12).

Immunofluorescence and ELISA confirmed the qPCR findings for Occludin, ZO-1, and Claudin-1 in the jejunum (Figure 5). The fluorescence intensity of Occludin, ZO-1, and Claudin-1 in the jejunum was notably decreased in group M relative to that in group N (*p* < 0.01). Conversely, the fluorescence intensity of Occludin, ZO-1, and Claudin-1 in the jejunum of group T was markedly greater than in that of group M (*p* < 0.05 or *p* < 0.01) (Figure 5A–F). Additionally, the results of ELISA showed that protein expression levels of ZO-1, Occludin, and Claudin-1 in the jejunum were markedly reduced in group M relative to those in group N (*p* < 0.05 or *p* < 0.01) (Figure 5G–I). In comparison to group M, group T showed significantly increased expression of Occludin, ZO-1, and Claudin-1 (*p* < 0.05), consistent with the immunofluorescence results.

### 3.6. Jejunal Oxidative Stress and Senescence Markers

**Figure 6:**
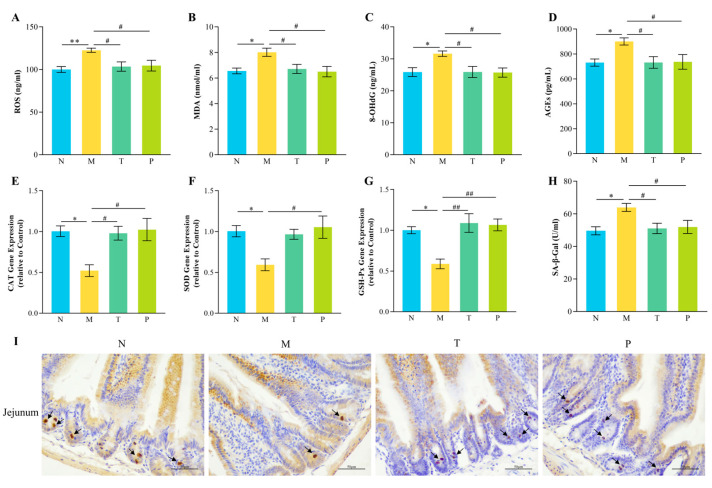
Results of oxidative and aging indexes in the mouse jejunum. (**A**) ROS; (**B**) MDA; (**C**) 8-OHdG; (**D**) AGEs; (**E**) CAT; (**F**) SOD; (**G**) GSH-Px; (**H**) SA-β-Gal; (**I**) Ki67 staining of the jejunum. “→”: proliferating cell; “^#^” denotes *p* < 0.05; “^##^” denotes *p* < 0.01; “*” denotes *p* < 0.05; “**” denotes *p* < 0.01 (*n* = 12).

As illustrated in Figure 6, the expression levels of Catalase (CAT), *Glutathione peroxidase* (*GSH-Px*), and *Superoxide dismutase* (*SOD*) in the jejunal tissues of group M were significantly diminished (*p* < 0.05) in comparison to those in group N, while the expression levels of ROS, 8-OHdG, MDA, AGEs, and SA-β-Gal activity were markedly elevated (*p* < 0.05 or *p* < 0.01). In addition, the quantity of proliferating cells in intestinal crypts was decreased in group M relative to that in group N. Relative to those in group M, the levels of ROS, 8-OHdG, MDA, AGEs, and SA-β-Gal were markedly diminished in group T (*p* < 0.05), while the gene expression of *CAT* and *GSH-Px* was significantly enhanced (*p* < 0.05 or *p* < 0.01). Concurrently, the quantity of proliferating cells in the intestinal crypts of the T and P groups rose in comparison with that in the M group.

### 3.7. Jejunal Inflammatory Factor Expression

**Figure 7:**
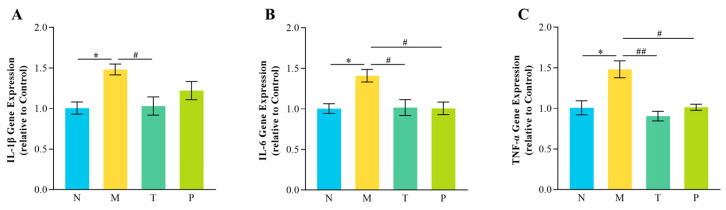
Expression of genes related to inflammatory factors in the mouse intestine. (**A**) IL-1β; (**B**) IL-6; (**C**) TNF-α. “^#^” denotes *p* < 0.05; “^##^” denotes *p* < 0.01; “*” denotes *p* < 0.05. (*n* = 3).

Figure 7 illustrates that, in comparison to group N, group M demonstrated significantly increased expression levels of IL-1β, TNF-α, and IL-6 in the jejunum (*p* < 0.05). In comparison to group M, group T showed significantly reduced expression levels of IL-1β, TNF-α, and IL-6 (*p* < 0.05 or *p* < 0.01), while group P demonstrated markedly decreased expression levels of IL-6 and TNF-α (*p* < 0.05).

### 3.8. RNA Sequencing and Functional Analysis

**Figure 8:**
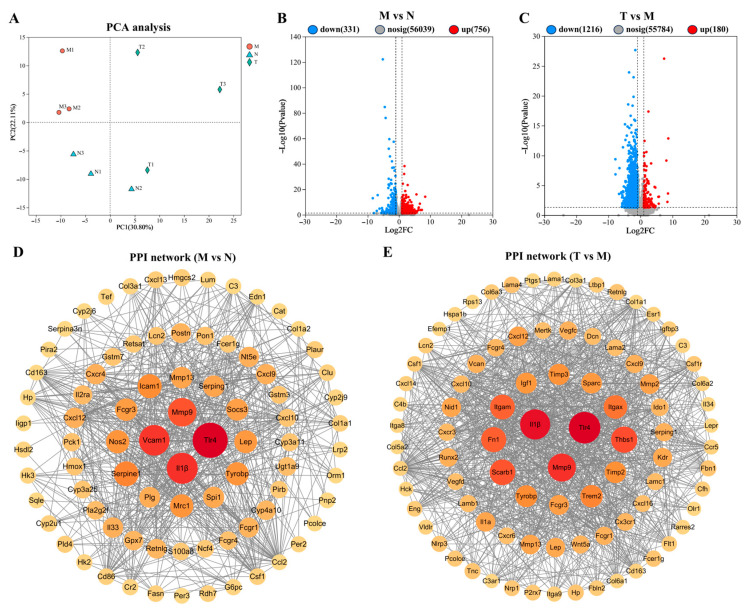
Effect of CPE on gene expression in the intestine of D-gal-administered mice. (**A**) PCA score plot; (**B**) group M vs. group N differentially expressed gene volcano plot; (**C**) group T vs. group M differentially expressed gene volcano plot; (**D**) group M vs. N PPI network; (**E**) group T vs. M PPI network. The size and color of nodes are set according to the degree. (*n* = 3).

Principal component analysis (PCA) results (Figure 8A) demonstrated a clear distinction between groups M and N, while the group T clustered closely with group N. Additionally, there were 1087 DEGs (756 up-regulated and 331 down-regulated) between groups M and N and 1396 DEGs (180 up-regulated and 1216 down-regulated) between groups T and M. Key DEGs ranked by degree between groups M and N included *TLR4*, *IL-1β*, *Vcam1*, and *Mmp9* (Figure 8D), while those between groups T and M were *TLR4*, *IL-1β*, and *Mmp9* (Figure 8E).

**Figure 9:**
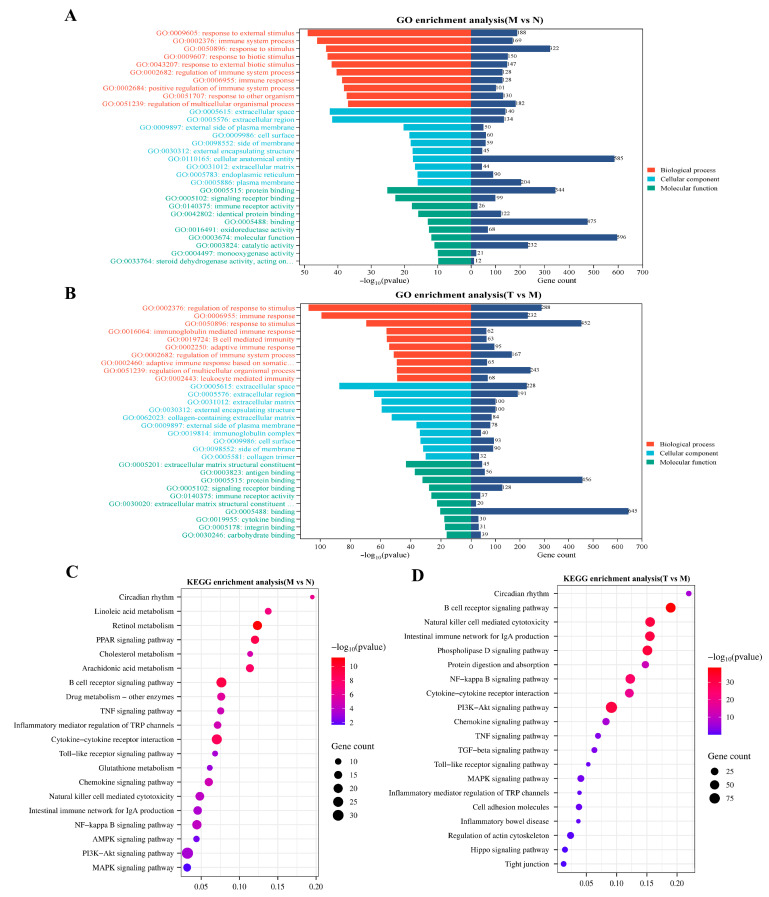
Differential gene function analysis. (**A**) Group M vs. group N GO functional enrichment analysis; (**B**) group T vs. group M GO functional enrichment analysis; (**C**) group M vs. group N KEGG pathway enrichment analysis; (**D**) group T vs. group M KEGG pathway enrichment analysis (*n* = 3).

DEGs were further analyzed using KEGG and GO enrichment analyses. For group M vs. group N, GO enrichment analysis revealed that in the biological process (BP) category, the DEGs were predominantly enriched in response to external stimulus, immune response, and immune system process. In the cellular component (CC) category, significant enrichment was observed in the extracellular space, external encapsulating structure, and extracellular region. In the molecular function (MF) category, these DEGs were primarily associated with oxidoreductase activity, signaling receptor binding, and immune receptor activity (Figure 9A). For group T vs. group M, GO enrichment analysis indicated that the DEGs were predominantly enriched in the immune response, regulation of response to stimulus, and regulation of immune system processes within the BP category. In the CC category, enrichment was observed in the extracellular region, external encapsulating structure, and cell surface. In the MF category, these DEGs were associated with extracellular matrix structural constituents, signaling receptor binding, and cytokine binding (Figure 9B). KEGG enrichment analysis revealed that the DEGs in group M vs. group N were markedly enriched in pathways related to as circadian rhythm, NF-κ, Toll-like receptor, TNF, AMPK, and MAPK (Figure 9C). For group T vs. group M, the DEGs were enriched in pathways related to NF-κB, tight junctions, Toll-like receptor, TNF, PI3K-Akt, and the intestinal immune network for IgA production (Figure 9D).

### 3.9. TLR4/NF-κB Pathway Protein Expression

**Figure 10:**
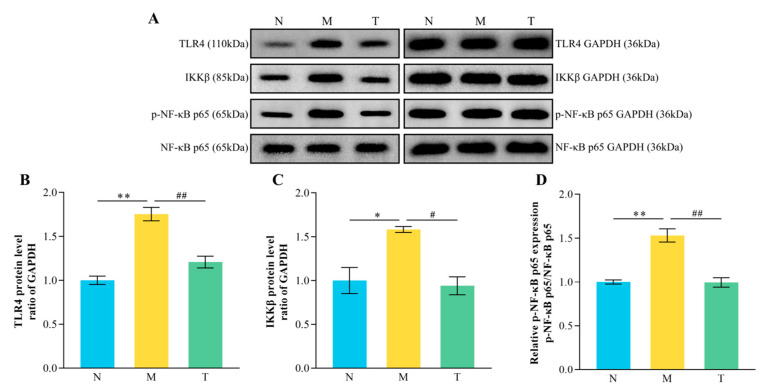
Regulatory effect of CPP on the TLR4/NF-κB signaling pathway. (**A**) TLR4, IKKβ, p-NF-κB, and NF-κB protein expression map; (**B**) TLR4 protein expression; (**C**) IKKβ protein expression; (**D**) NF-κB p65/p-NF-κB p65 protein expression. “^#^” denotes *p* < 0.05; “^##^” denotes *p* < 0.01; “*” denotes *p* < 0.05; “**” denotes *p* < 0.01 (*n* = 3).(Figure S1).

Western blotting results (Figure 10) indicated that, in comparison to group N, group M exhibited markedly increased expression levels of TLR4 and IKKβ proteins, as well as an elevated NF-κB p65/p-NF-κB p65 protein ratio (*p* < 0.05 or *p* < 0.01). In contrast, group T showed markedly reduced levels of TLR4 and IKKβ proteins and a lower NF-κB p65/p-NF-κB p65 protein ratio in comparison to group M (*p* < 0.05 or *p* < 0.01), further supporting the protective role of CPP in modulating this pathway.

## 4. Discussion

### 4.1. Effect of CPP on Body Weight of Mice

D-gal disrupts intestinal villus morphology, reducing nutrient absorption efficiency and leading to body weight loss in mice [21]. This study demonstrated that the intraperitoneal injection of D-gal led to a reduced rate of body weight gain, with the final body weight in group M substantially lower than that in group N. CPP treatment significantly alleviated the body weight loss, resulting in higher body weights compared to those in the M group. Interestingly, CPP’s efficacy surpassed that of Vit C administration. This effect may be attributed to CPP’s composition of small bioactive peptides, which are easily digestible and absorbable. These peptides provide essential amino acids, promote gastrointestinal motility, and stimulate hormone secretion [25]. For instance, collagen polypeptide from tilapia skin has been reported to increase body weight in D-gal-induced senescent mice [26], aligning with the findings of this study.

### 4.2. Effect of CPP on Intestinal Tissue Structure in Mice

Intestinal mucosal morphology is a critical marker of nutrient absorption capacity and barrier function [27]. Goblet cells and their secreted mucins are essential components of the intestinal barrier, protecting the mucosa from harmful substances [28]. In this study, D-gal administration led to villus atrophy, disorganized arrangement, epithelial loss, and a reduction in goblet cells. Additionally, villus length and V/C ratio were significantly decreased, while crypt depth was significantly increased. These findings align with previous reports [21,22,29], confirming the deleterious effects of D-gal on intestinal health. Peptides have been shown to repair intestinal mucosa, enhance crypt secretory function, and promote villus growth [30]. CPP, in particular, has been demonstrated to ameliorate intestinal structural damage caused by immunosuppression and to increase mucin gene expression levels [17]. This study demonstrated that mice treated with CPP showed considerable enhancements in intestinal villus morphology, marked by increased villus length and V/C ratio, reduced crypt depth, and an increase in goblet cell numbers. These findings suggest that CPP improves nutrient absorption and barrier function by repairing D-gal-induced intestinal mucosal damage, supporting the observed increase in body weight with CPP treatment.

### 4.3. Effect of CPP on Intestinal Barrier Function in Mice

TJs, such as ZO-1, Occludin, and Claudin-1, are crucial for preserving intestinal barrier integrity [31]. DAO and LPS levels in the serum are reliable indicators of intestinal barrier damage; elevated levels reflect increased permeability and inflammation [32,33]. The results of this study demonstrate that D-gal administration resulted in significant damage to intestinal tissue morphology in mice, evidenced by increased serum DAO activity and LPS levels, along with reduced expression of ZO-1, Occludin, and Claudin-1 in the intestine, indicating the disruption of the intestinal barrier. Bioactive peptides, as essential nutrients, can enhance intestinal barrier function by promoting protein synthesis, thereby providing energy for the production of TJ proteins [34]. For instance, Tenebrio molitor peptides have been shown to reduce serum DAO activity and LPS levels, augment the expression of ZO-1 and Occludin, and mitigate radiation-induced intestinal barrier damage [35]. Similarly, CPP has been reported to enhance expression of *ZO-1*, *Occludin*, and *Claudin-1* genes, thereby strengthening intestinal barrier function in immunosuppressed mice [17]. The findings of this study indicated that serum levels of DAO activity and LPS were diminished in the CPP-treated group of mice, and the expression of Occludin, Claudin-1, and ZO-1 in the jejunum was normalized, akin to the effects observed with vitamin C. In summary, CPP can effectively alleviate D-gal-induced intestinal barrier impairment by augmenting the expression of TJs.

### 4.4. Effect of CPP on Indicators Related to Intestinal Oxidation and Aging in Mice

The intestine is highly sensitive to oxidative stress and is one of the primary target organs of ROS attacks [36,37,38]. Factors such as ROS, 8-OHdG, MDA, GSH-Px, CAT, and SOD are commonly employed to assess particular biomarkers of oxidative damage [39,40,41]. Persistent oxidative stress can drive cellular senescence, leading to elevated levels of senescence markers such as AGEs and SA-β-Gal in tissues and organs [19]. Additionally, the number of proliferating cells in the intestinal crypts decreases under aging conditions [42]. The findings of this study indicated that in group M, mice exhibited increased levels of intestinal ROS, MDA, AGEs, 8-OHdG, and SA-β-Gal, along with decreased mRNA expression of antioxidant enzymes and a reduced number of Ki67-positive proliferating cells. These findings indicate that D-gal induced oxidative damage and aging in the intestinal tissue of mice. Urolithin B has been shown to reduce intestinal AGEs and MDA levels in D-gal-treated mice while increasing levels of SOD, GSH-Px, and CAT, thereby alleviating oxidative stress-induced intestinal aging [22]. Our previous research demonstrated that CPP exhibits potent antioxidant properties, enhances antioxidant enzyme activity and the number of Ki67-positive hepatocytes, and mitigates D-gal-induced liver aging in mice [16]. In this study, on the one hand, CPP reduced intestinal ROS, 8-OHdG, and MDA levels while increasing the gene expression of *CAT* and *GSH-Px*, thereby suppressing ROS-induced lipid and nucleic acid damage and alleviating oxidative stress in the intestine. On the other hand, CPP decreased AGE and SA-β-Gal levels and enhanced the number of Ki67-positive proliferative cells in the intestinal crypts, indicating that CPP alleviates intestinal aging by mitigating oxidative damage in the intestine.

### 4.5. Effect of CPP on TLR4/NF-κB Signaling Pathway in Mouse Intestines

Aging leads to a decline in intestinal immune function, triggering inflammatory responses and compromising the intestinal barrier [43,44,45]. The overexpression of Mmp9 disrupts intestinal tissue structure, reduces the number of goblet cells, and increases levels of inflammatory cytokines, thereby exacerbating intestinal inflammation [46]. IL-1β, a key cytokine in intestinal inflammation, is closely associated with the disruption of intestinal barrier function [47]. TLR4, an essential element of the innate immune response, is vital in immunological activation [48]. A PPI network analysis of the DEGs obtained from the transcriptome identified *TLR4*, *IL-1β*, and *Mmp9* as key targets of CPP. Additionally, GO terms related to immune-associated biological processes and molecular functions were significantly enriched, suggesting that CPP alleviates D-gal-induced immune dysfunction and inflammatory responses in the mouse intestine. Signaling pathways such as NF-κB, mTOR, AMPK, and ROCK/MLCK play crucial roles in regulating intestinal barrier function [49,50]. In this study, KEGG enrichment analysis revealed significant enrichment of the NF-κB and AMPK signaling pathways, with particular emphasis on the NF-κB signaling pathway. Notably, upstream and downstream pathways related to NF-κB, such as TLR receptor and TNF signaling pathways, were also significantly enriched. Peptide-based substances have been reported to protect the intestinal barrier via the NF-κB signaling pathway [51]. Therefore, the TLR4/NF-κB signaling pathway was selected for further validation in this study. Additionally, the enrichment of the TJ pathway aligns with the observed increased expression of intestinal TJs in the CPP-treated group, further supporting the protective role of CPP in alleviating D-gal-induced intestinal barrier damage in aging mice.

NF-κB is the terminal transcription factor of the TLR4 signaling pathway and, upon phosphorylation, translocates to the nucleus and binds to DNA to activate the expression of inflammatory cytokines such as IL-1β, IL-6, and TNF-α [52]. These inflammatory cytokines suppress the production of TJs, thereby compromising the intestinal barrier [47,53,54]. IKKβ promotes the phosphorylation and degradation of IκB through phosphorylation at Ser177 and Ser181, subsequently activating the NF-κB transcription factor [55]. Egg white mucin hydrolysate inhibits the lipopolysaccharide-induced inflammatory response and increases permeability in Caco-2 cells by regulating the NF-κB signaling pathway and restoring the expression of TJs between cells [56]. Similarly, buffalo milk peptides can ameliorate inflammation-induced intestinal barrier damage via NF-κB signaling pathway regulation [57]. In this study, the administration of CPP significantly down-regulated the elevated expression levels of TLR4, IKKβ, and p-NF-κB p65 proteins induced by D-gal administration. This aligns with the observed reduction in the mRNA expression of the jejunal inflammatory cytokines *IL-1β*, *IL-6*, and *TNF-α* following CPP treatment. These findings suggest that CPP mitigates inflammatory responses by regulating the TLR4/NF-κB signaling pathway, thereby protecting against intestinal barrier damage.

## 5. Conclusions

**Figure 11:**
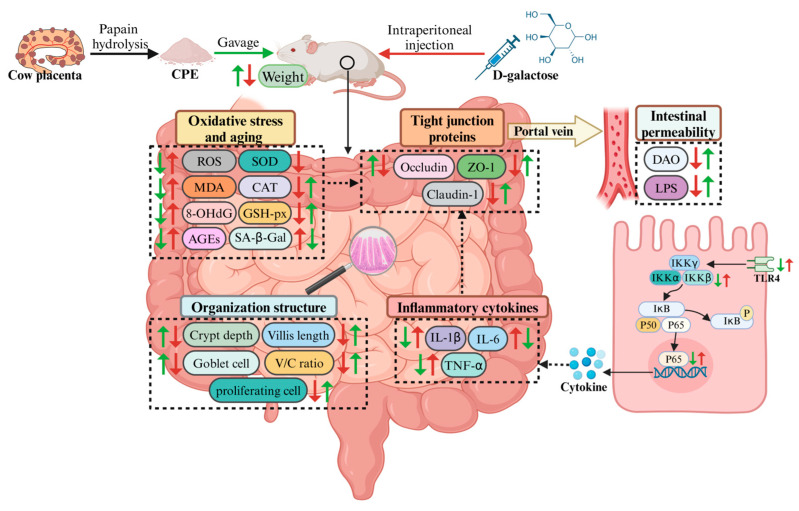
Protective effects of CPP on intestinal barrier damage in D-gal-induced aged mice. Green arrows denote the impact of CPP treatment, while red arrows signify the effect of D-gal administration on mice. ↑: up-regulation; ↓: down-regulation (This figure was created using BioRender.com).

This study demonstrates the protective effects of CPP on intestinal barrier damage in D-gal-induced aged mice. CPP effectively alleviates structural damage and dysfunction in intestinal tissue by enhancing antioxidant defenses, inhibiting the TLR4/NF-κB signaling pathway, and diminishing inflammatory responses. These actions promote TJ expression, decrease intestinal permeability, and counteract the detrimental effects of aging on intestinal barrier integrity (Figure 11). In conclusion, CPP emerges as a promising natural therapeutic agent, offering an effective intervention strategy for addressing aging-related intestinal barrier dysfunction and contributing to broader anti-aging applications.

## Data Availability

All sequencing data can be accessed via the NCBI Sequence Read Archive with the accession number PRJNA1190606.

## Supplementary Materials

The following supporting information can be downloaded at: https://www.mdpi.com/article/10.3390/vetsci12030229/s1. Table S1: List of primers used for Q-PCR analysis; Table S2: List of antibodies used for immunofluorescence and immunohistochemistry; Table S3: List of antibodies used for Western blot analysis. Figure S1: Original western blot for three repeats.
