# Supplementary material for: Cow Placenta Peptides Ameliorate D-Galactose-Induced Intestinal Barrier Damage by Regulating TLR/NF-κB Pathway
Source: Vet Sci. 2025 Mar 3;12(3):229. doi: 10.3390/vetsci12030229 (PMC11945863; doi:10.3390/vetsci12030229)
Supplement: Supplementary file 1 [file vetsci-12-00229-s001.zip › vetsci-3459380-supplementary.pdf]

Table S1. List of primers used for Q-PCR analysis

Gene	Forward primers (5′-3′)	Reverse primers (5′-3′)	Size (bp)
CAT	AGCGACCAGATGAAGCAGTG	TCCGCTCTCTGTCAAAGTGTG	181
GSH-Px	AGTCCACCGTGTATGCCTTCT	GAGACGCGACATTCTCAATGA	105
SOD	AACCAGTTGTGTTGTCAGGAC	CCACCATGTTTCTTAGAGTGAGG	139
IL-1β	GCAACTGTTCCTGAACTCAACT	ATCTTTTGGGGTCCGTCAACT	89
IL-6	TAGTCCTTCCTACCCCAATTTCC	TTGGTCCTTAGCCACTCCTTC	76
TNF-α	CCGGGAGAAGAGGGATAGCTT	TCGGACAGTCACTCACCAAGT	113
Claudin-1	GCCTTGATGGTAATTGGCATCC	GGCCACTAATGTCGCCAGAC	165
Occludin	TTGAAAGTCCACCTCCTTACAGA	CCGGATAAAAAGAGTACGCTGG	189
ZO-1	GCTTTAGCGAACAGAAGGAGC	TTCATTTTTCCGAGACTTCACCA	156
β-actin	GGCTGTATTCCCCTCCATCG	CCAGTTGGTAACAATGCCATGT	154

Table S2. List of antibodies used for immunofluorescence and immunohistochemistry

Antibody	Dilution	Source	batch number
Claudin-1	1:200	Zen-bioscience	343203
Occludin	1:500	Proteintech	27260-1-AP
ZO-1	1:500	Proteintech	21773-1-AP
Ki67	1:2000	Huabio	HA721115

Table S3. List of antibodies used for western blot analysis

Antibody	Dilution	Source	batch number
GAPDH	1:5000	Zen-bioscience	200306-7E4
TLR4	1:8000	Proteintech	66350
IKKβ	1:1000	Abcam	ab124957
NF-KB p65	1:10000	Abcam	ab32536
P-NF-KB p65	1:1000	Abcam	ab76302

TLR4-Repeat 1

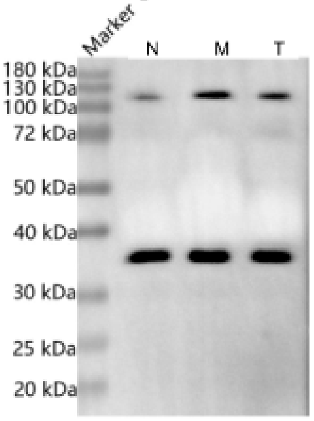

TLR4,100-110kDa+GAPDH,36kDa

TLR4-Repeat 2

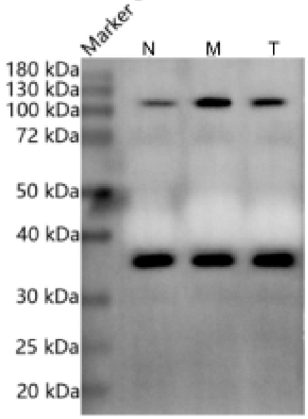

TLR4,100-110kDa+GAPDH,36kDa

TLR4-Repeat 3

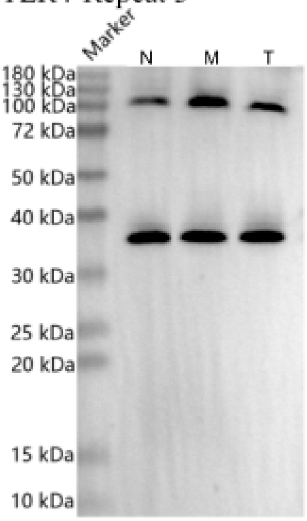

TLR4 ,100-110kDa+GAPDH,36kDa

IKKβ-Repeat 1

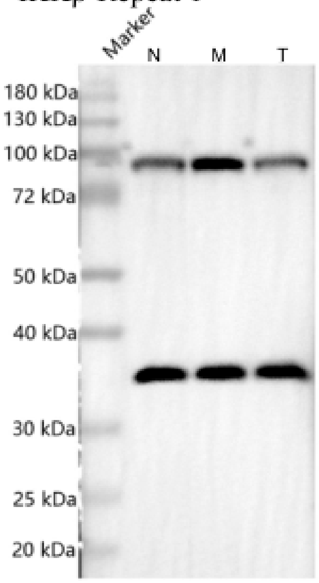

IKKβ,85kDa+GAPDH,36kDa

IKKβ-Repeat 2

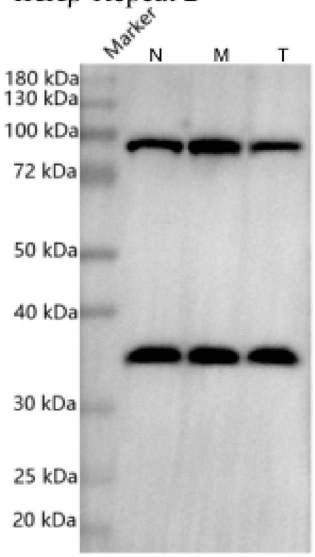

IKKβ,85kDa+GAPDH,36kDa

IKKβ-Repeat 3

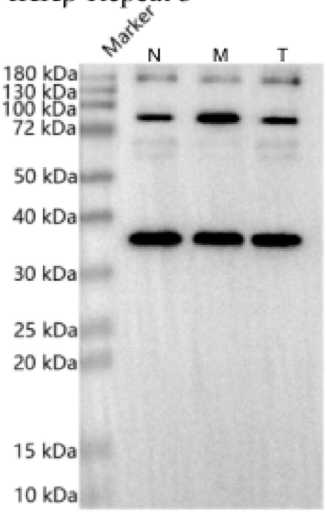

IKKβ,85kDa+GAPDH,36kDa

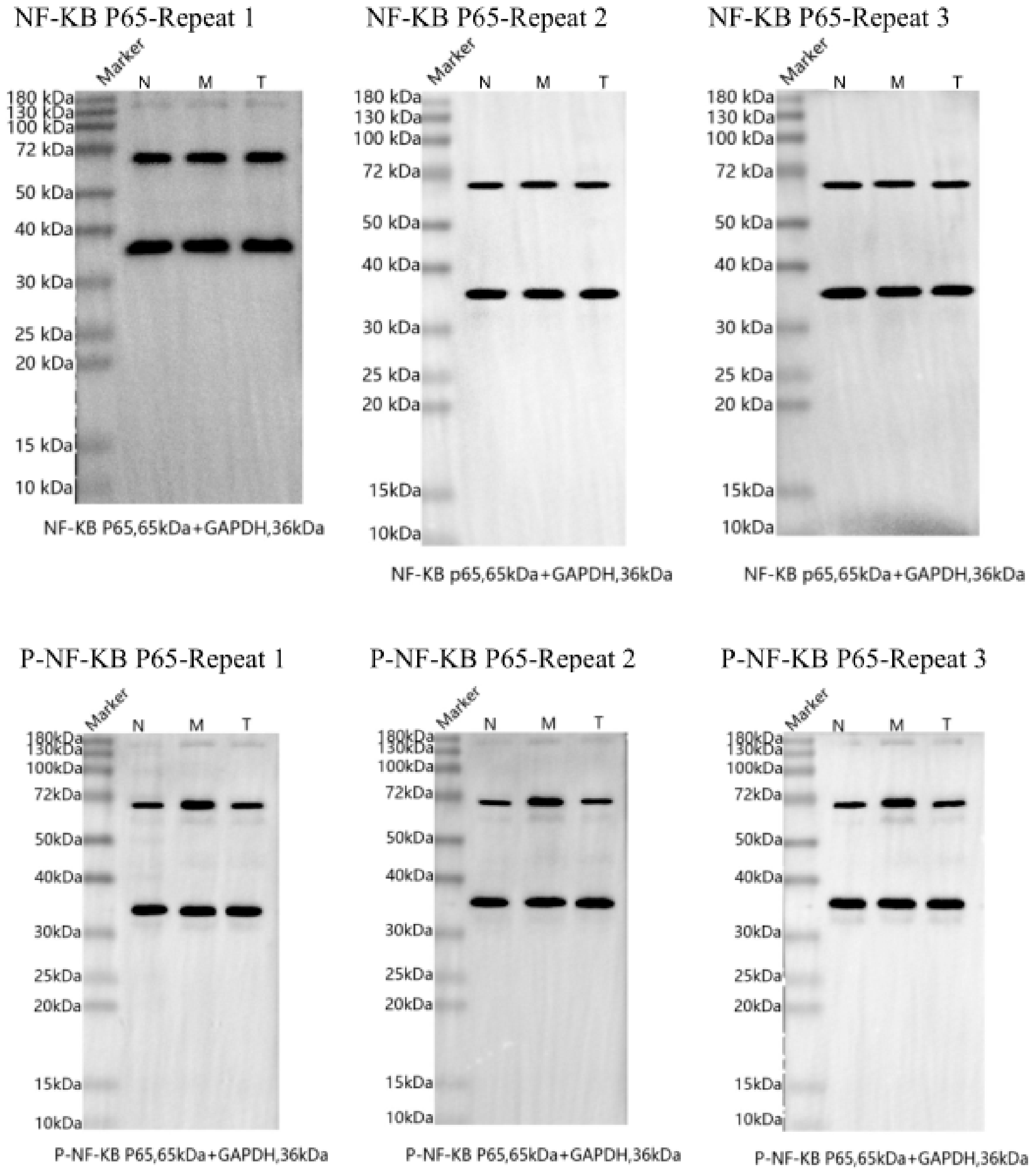

Figure S1 Original western blot for three repeats
